# LncRNA LINC00667 gets involved in clear cell renal cell carcinoma development and chemoresistance by regulating the miR-143-3p/ZEB1 axis

**DOI:** 10.18632/aging.205029

**Published:** 2023-10-11

**Authors:** Jianjun Zhao, Pengjie Chen, Chao Tan, Xiaolong Cheng, Weichuan Zhang, Chong Shen, Dongli Zhang

**Affiliations:** 1 Department of Urology, Affiliated Hospital of Hebei Engineering University, Handan 056002, Hebei, China; 2 Department of Geriatrics, Handan Central Hospital, Handan 056001, Hebei, China

**Keywords:** clear cell renal cell carcinoma, LINC00667, progression, miR-143-3p, ZEB1

## Abstract

Background: Clear cell renal cell carcinoma (ccRCC) is identified as a malignant tumor in the urinary tract. The research was an attempt to probe the biological function and molecular mechanism of lncRNA LINC00667 in ccRCC development.

Methods: qRT-PCR monitored LINC00667, miR-143-3p, and ZEB1 levels. The models of LINC00667, miR-143-3p, and ZEB1 overexpression or knockdown were constructed in ccRCC cells. Cell proliferation, apoptosis, migration, and invasion of the cells were detected. The levels of apoptosis-associated proteins and epithelial-mesenchymal transition (EMT)-related proteins, and ZEB1 were detected by WB. Dual-luciferase reporter assay and RNA pull-down assay identified the binding association between LINC00667 and miR-143-3p, miR-143-3p and ZEB1. Moreover, a xenograft tumor model in nude mice was used for evaluating tumor growth *in vivo*.

Results: LINC00667 and ZEB1 displayed high expression in ccRCC tissues and cells. miR-143-3p was lowly expressed in ccRCC tissues and cells. LINC00667 targeted and repressed miR-143-3p, which inhibited ZEB1 expression in a targeted manner. Overexpression of LINC00667 facilitated ccRCC cell proliferation, migration, invasion and EMT and retarded apoptosis, whereas LINC00667 knockdown or miR-143-3p overexpression exerted reverse effects. The rescue experiments indicated that overexpressing miR-143-3p dampened LINC00667-mediated oncogenic effects. Overexpressing ZEB1 diminished miR-143-3p-mediated tumor-suppressive effects. *In-vivo* experiments displayed that overexpression of LINC00667 contributed to the tumor growth of ccRCC cells, in contrast to miR-143-3p overexpression, which restrained the tumor growth.

Conclusions: LINC00667 is up-regulated in ccRCC and enhances the ZEB1 expression by targeting miR-143-3p, which in turn accelerates ccRCC progression and induces chemoresistance.

## INTRODUCTION

Clear cell renal cell carcinoma (ccRCC), among the most aggressive malignancies of the genitourinary system common in adults, makes up around 3% of grown-up malignant tumors [1, 2]. At this stage, surgical resection remains the mainstay of treatment for ccRCC, while the relapse and metastasis rate reach as high as 30-35% of patients who undergo nephrectomy [3]. Achieving early diagnosis and effective therapy of ccRCC has become the main goal of ccRCC management [4]. Molecular markers, such as CAIX, PTEN, and CXCR4, have been found to show altered gene expression, gene mutations, and methylation status, and they provide more treatment selections in ccRCC [5], suggesting that investigating novel biomarkers for the early diagnosis, individualized treatment, and prognostic determination of ccRCC is necessary.

Non-coding RNAs (ncRNAs) belong to large group RNAs without protein-coding abilities. They have a length longer than 200 nt. Though lncRNAs nearly cannot encode proteins, they modulate the profiles of genes at epigenetic, transcriptional, and post-transcriptional levels through their interaction with DNAs, proteins, as well as RNAs, and contribute to tumor development, neuroscience, and many other biological fields [6–8]. Recent studies have revealed that lncRNAs are associated with kidney cancer development [9–11]. lncRNA LINC00667, a promoter for umpteen tumors, presents a high-level expression in colorectal cancer cells. Meanwhile, the LINC00667/miR-449b-5p/YY1 axis boosts colorectal cancer cell proliferation and migration [12]. Besides, LINC00667 contributes to the malignant progression of nephroblastoma [13], hepatocellular carcinoma (HCC) [14], and cholangiocarcinoma [15].

According to ceRNA regulatory theory, lncRNAs can bind to miRNAs as ceRNAs and attenuate the regulatory impact of miRNAs on the targeted genes. The basic mechanism of action is that RNA can bind to MREs. Different RNAs can interact competitively with the same miRNAs, thus forming a complex regulatory network that affects tumor growth and development. WU et al. [16] discovered that SNHG12 sponged miR-129-5p and modulated the expression of the MDM4 and p53 pathways during ccRCC development. Studies regarding the influence of lncRNAs on ccRCC via ceRNA patterns are scarce [17, 18]. Therefore, the construction of ceRNA regulatory networks and the search for lncRNAs in the network that inextricably pertain to ccRCC prognosis and have important biological functions will help the diagnosis and treatment of ccRCC.

Presently, we tested LINC00667 level alterations in renal cancer cells and ccRCC tumor tissues. Overexpressed LINC00667 was observed in the tumor cells and tissues. Furthermore, we conducted bioinformatic analysis and found that LINC00667 potentially targets miR-143-3p, and ZEB1 is a target of miR-143-3p. Interestingly, both miR-143-3p and ZEB1 were altered in renal cancer cells and tissues. We therefore speculated that the ceRNA regulatory network of lncRNA LINC00667-miR-143-3p-ZEB1 presumably partook in the modulation of ccRCC evolvement. Moreover, we investigated this axis in the malignant phenotypes and sensitivity to cisplatin of human renal cancer cell lines. We hope to provide new ideas and therapeutic targets for ccRCC.

## RESULTS

### Alterations in LINC00667, miR-143-3p, and ZEB1 levels in ccRCC tissues and cells

**Figure 1:**
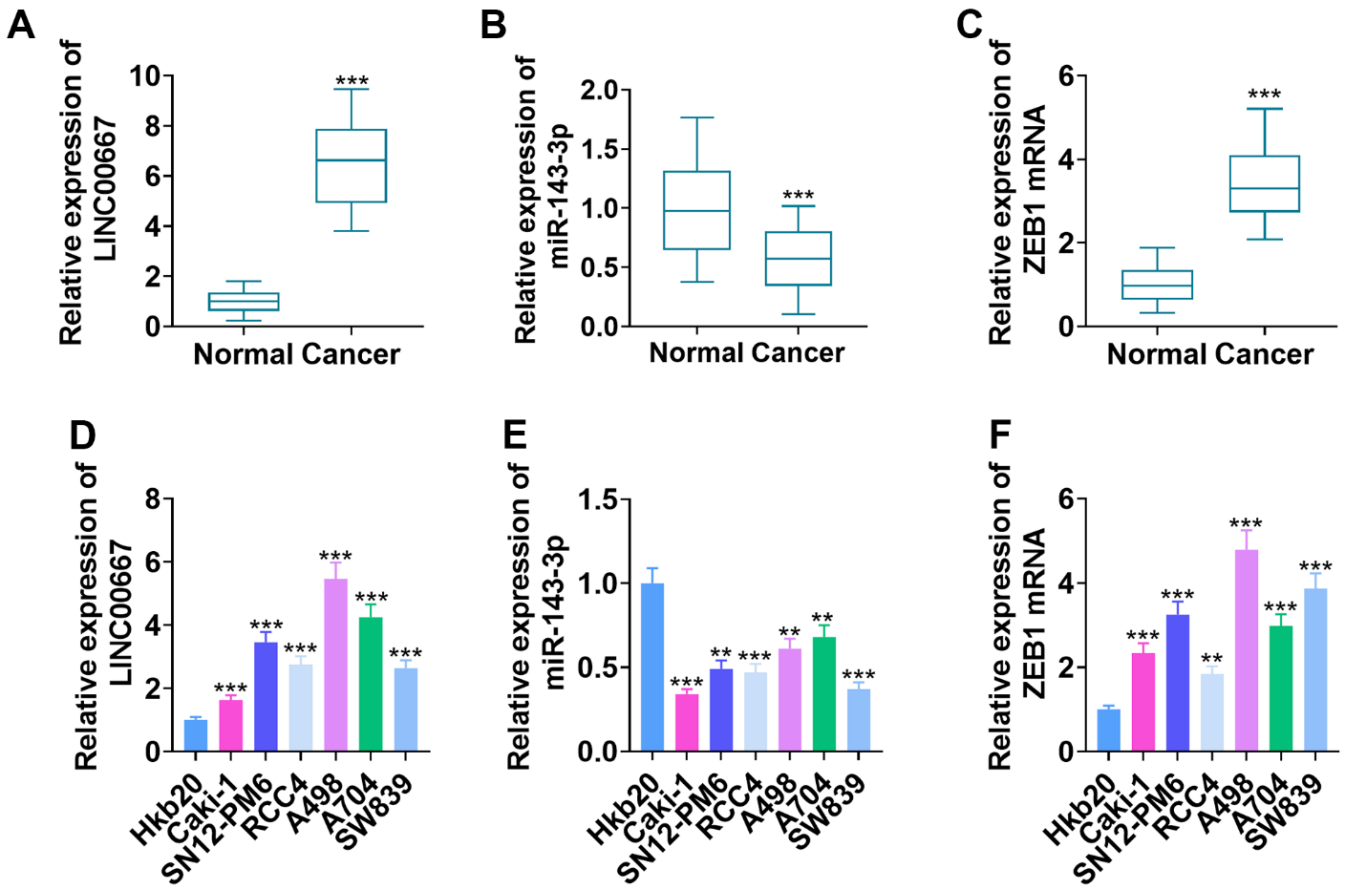
**Changes in LINC00667, miR-143-3p, and ZEB1 levels in ccRCC tissues and cells.** (**A**–**C**) LINC00667, miR-143-3p, and ZEB1 levels in the cancerous (N=52) and normal (N=52) tissues of ccRCC patients measured by qRT-PCR (***P<0.001, versus Normal). (**D**–**F**) LINC00667, miR-143-3p and ZEB1 levels in Hkb20, Caki-1, SN12-PM6, RCC4, A498, A704, and SW839 probed by qRT-PCR (**P<0.01, ***P<0.001, vs. Hkb20).

qRT-PCR ascertained LINC00667, miR-143-3p, and ZEB1 mRNA profiles in the cancerous and non-tumor tissues of ccRCC patients. Consequently, LINC00667 and ZEB1 mRNA levels increased, whereas miR-143-3p level was lowly expressed in tumor tissues versus normal tissues (Figure 1A–1C, *P*<0.05). In parallel, LINC00667 and ZEB1 mRNA levels were markedly facilitated, and the miR-143-3p level was vigorously abated in Caki-1, SN12-PM6, RCC4, A498, A704, and SW839 compared to human renal epithelial cells Hkb20 (Figure 1D–1F, *P*<0.05). From our collection of ccRCC samples, it was observed that high levels of LINC00667 had no significant relations to the age and gender of patients with ccRCC but showed a marked association with T stage (p=0.017) and Furhman pathological classification (p=0.042) (Table 1).

**Table 1:** The association between the LINC00667 level and clinical parameters.

**Characteristics**	**Samples**	**High (n=26)**	**Low (n=26)**	***P* value**
Age				0.780
≥60	23	12	11	
<60	29	14	15	
Gender				0.243
Male	34	19	15	
Female	18	7	11	
T staging				
1+2	41	17	24	0.017*
3	11	9	2	
Fuhrman staging				0.042*
I+ II	45	20	25	
III+IV	7	6	1	

Note: *P<0.05.

### Functional effects of LINC00667 overexpression on ccRCC cells

**Figure 2:**
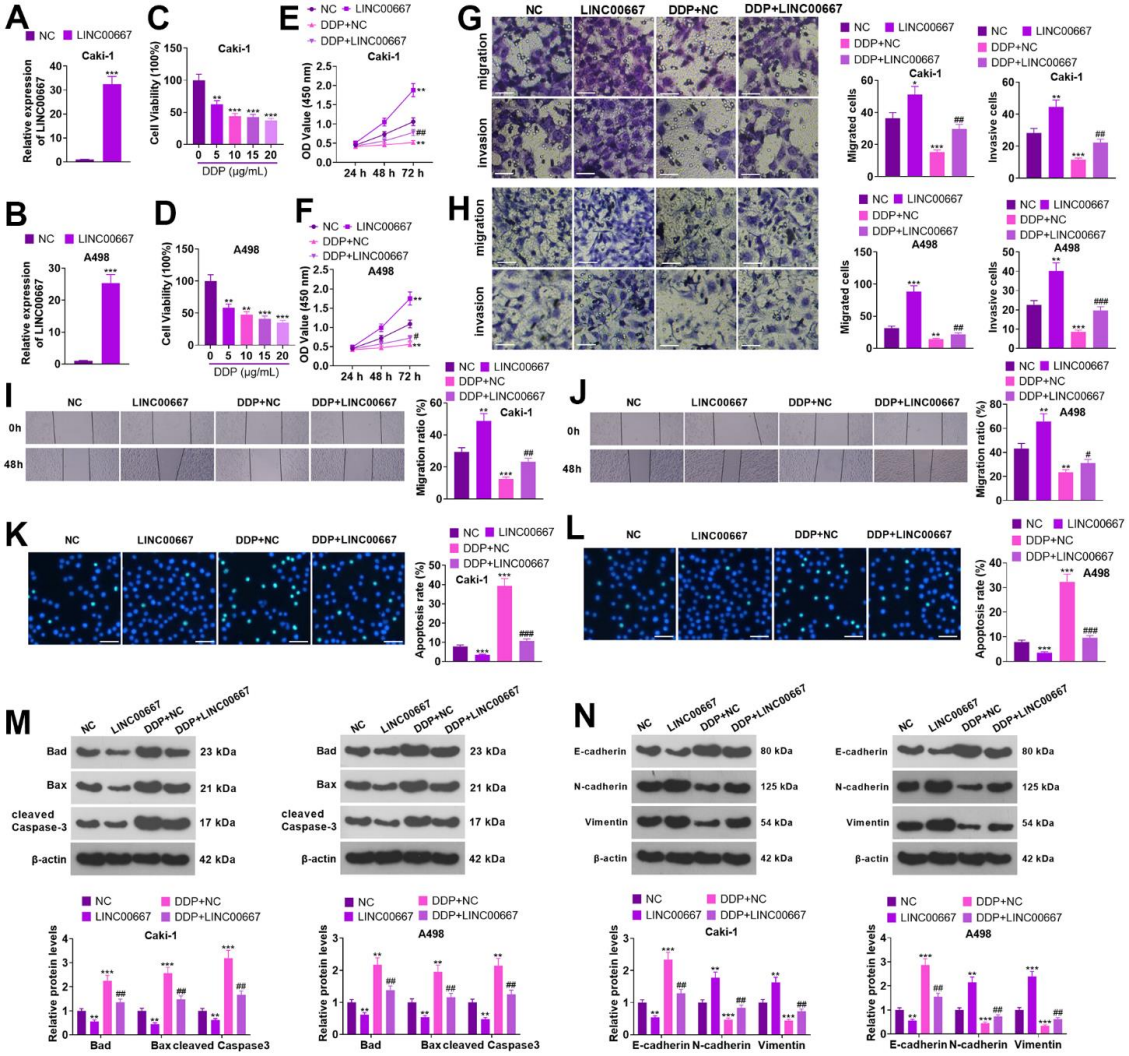
**Effects of overexpression of LINC00667 on ccRCC cells.** (**A**, **B**) Caki and A498 were transfected together with LINC00667 overexpression plasmids. The transfection validity of LINC00667 was checked by qRT-PCR. (**C**, **D**) DDP (0, 5, 10, 15, 20 μg/mL) treated Caki and A498 cells for 48 hours. Cell viability was measured through MTT. Caki and A498 cells transfected with LINC00667 overexpression plasmids were treated with DDP (5μg/mL) for 48 hours. (**E**, **F**) Cell viability examined through MTT. (**G**, **H**) The migration and invasion activities of the cells monitored through Transwell. Scale bar=100 μm. (**I**, **J**) Cell migration evaluated by the wound healing test. (**K**, **L**) Apoptosis gauged through TUNEL. Scale bar=50 μm. (**M**) Bad, Bax, and cleaved Caspase-3 levels determined via WB. (**N**) EMT assessed through E-cadherin, N-cadherin, and Vimentin detection. **P<0.01, ***P<0.001 (vs. NC). #*P*<0.05, ##*P*<0.01 (vs. DDP+NC). N=3.

Caki and A498 cells were transfected with LINC00667 overexpression plasmids or negative control (NC), respectively. The transfection efficiency was confirmed by the qRT-PCR (Figure 2A, 2B, *P*<0.05). DDP (0, 5, 10, 15 and 20 μg/mL) treated Caki and A498 for 48 hours. MTT revealed that the viability of Caki and A498 cells decreased with increasing DDP concentration (Figure 2C, 2D, *P*<0.05). DDP (5 μg/mL) was used for treating Caki and A498 cells with LINC00667 overexpression. MTT and Transwell assays showed that overexpressing LINC00667 remarkably boosted the viability, migration, and invasion of Caki and A498 cells (versus the NC group). DDP resulted in the reverse consequences, while Caki and A498 cells’ viability, migration, and invasion were dramatically repressed by overexpression of LINC00667 (Figure 2E–2J, *P*<0.05 versus the DDP+NC group). TUNEL assay and western blot indicated that DDP resulted in elevated apoptosis in Caki and A498 (vis-à-vis the NC group). Overexpressing LINC00667 remarkably restrained apoptosis (against NC or DDP+NC) (Figure 2K–2M, *P*<0.05). Compared to the NC group, DDP distinctly facilitated the levels of E-cadherin but retarded N-cadherin and Vimentin levels. The profile of E-cadherin was markedly reduced, whereas N-cadherin and Vimentin profiles were elevated after overexpression of LINC00667 versus the NC or DDP+NC group (Figure 2N, *P*<0.05). Thus, overexpression of LINC00667 boosted ccRCC cells’ viability, migration, and invasion, suppressed apoptosis, and attenuated ccRCC cells’ sensitivity to DDP.

### Functional effects of LINC00667 knockdown on ccRCC cells

**Figure 3:**
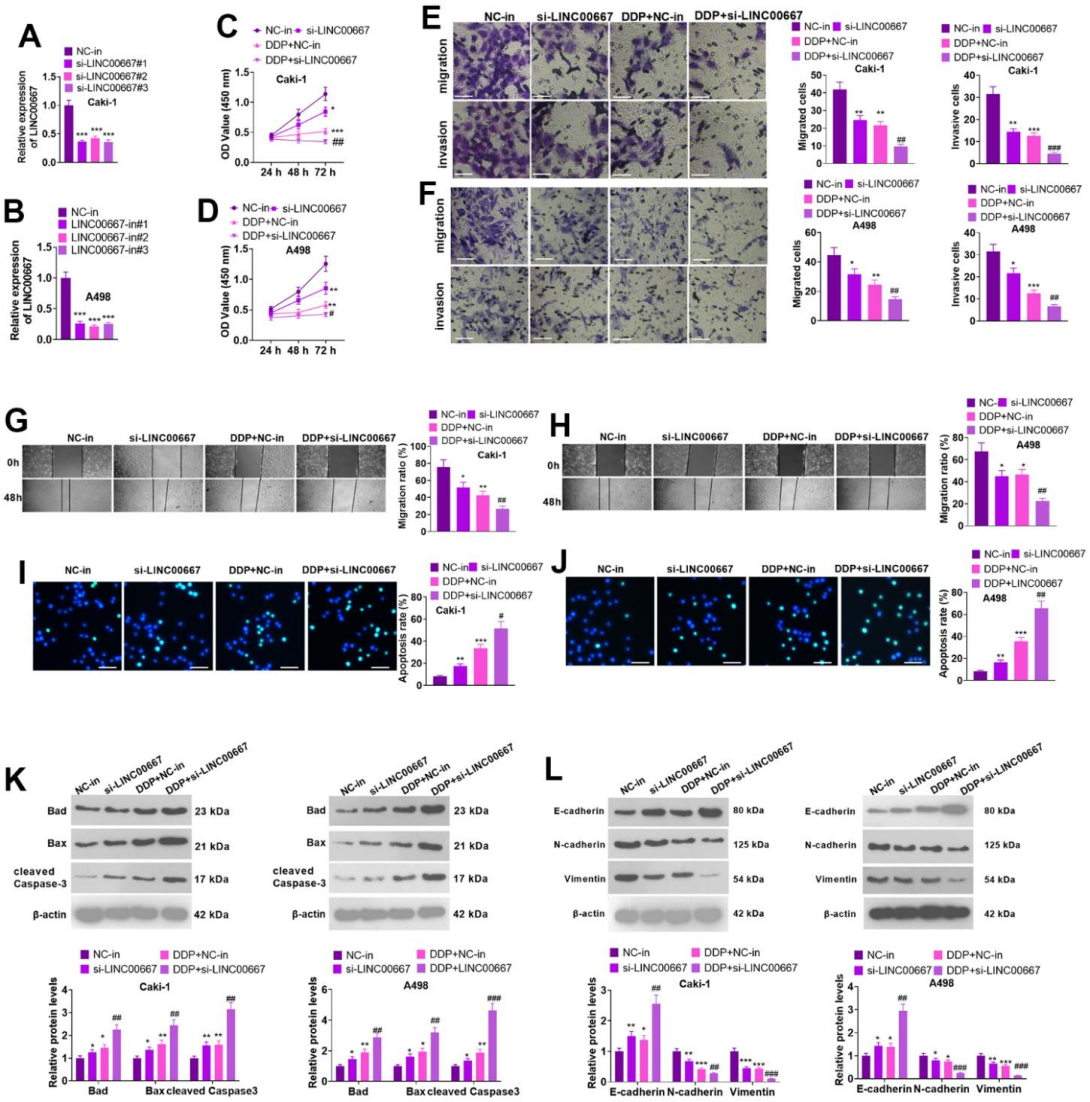
**Effects of overexpression of LINC00667 on ccRCC cells.** (**A**, **B**) Caki and A498 were transfected together with si-LINC00667 or NC-in. The transfection validity of LINC00667 was checked by qRT-PCR. (**C**, **D**) Cell viability was measured through MTT assay. Caki and A498 cells transfected with si-LINC00667 or NC-in, and then were treated with DDP (5μg/mL) for 48 hours. (**E**, **F**) The migration and invasion of the cells were monitored through Transwell. Scale bar=100 μm. (**G**, **H**) Cell migration was evaluated by the wound healing test. (**I**, **J**) Apoptosis was gauged through TUNEL assay. Scale bar=50 μm. (**K**) Bad, Bax, and cleaved Caspase-3 levels were determined via WB. (**L**) EMT was assessed by determining E-cadherin, N-cadherin, and Vimentin alterations. *P<0.05, **P<0.01, ***P<0.001 (vs. NC). #*P*<0.05, ##*P*<0.01, ###*P*<0.001 (vs. DDP+NC). N=3.

Caki and A498 cells were transfected with si-LINC00667 for knocking down LINC00667. The transfection efficiency was confirmed by the qRT-PCR (Figure 3A, 3B, *P*<0.05). Caki and A498 cells with LINC00667 knockdown or DDP treatment showed reduced cell viability, migration, and invasion. In addition, LINC00667 knockdown further increased the inhibitive effects of DDP on the two cells (Figure 3C–3H, *P*<0.05 versus the DDP+NC group). TUNEL assay and western blot indicated that LINC00667 knockdown and DDP administration both resulted in elevated apoptosis in Caki and A498 cells (vis-à-vis the NC-in group). si-LINC00667 remarkably accelerated the apoptotic level of Caki and A498 cells (against DDP+NC-in) (Figure 3I–3K, *P*<0.05). Moreover, the profile of E-cadherin was markedly enhanced, whereas N-cadherin and Vimentin profiles were reduced after the suppression of LINC00667 in DDP-treated cells (Figure 3L, *P*<0.05). Consequently, LINC00667 knockdown boosted ccRCC cells’ apoptosis and enhanced ccRCC cells’ sensitivity to DDP.

### Regulation of ccRCC tumor growth by LINC00667

**Figure 4:**
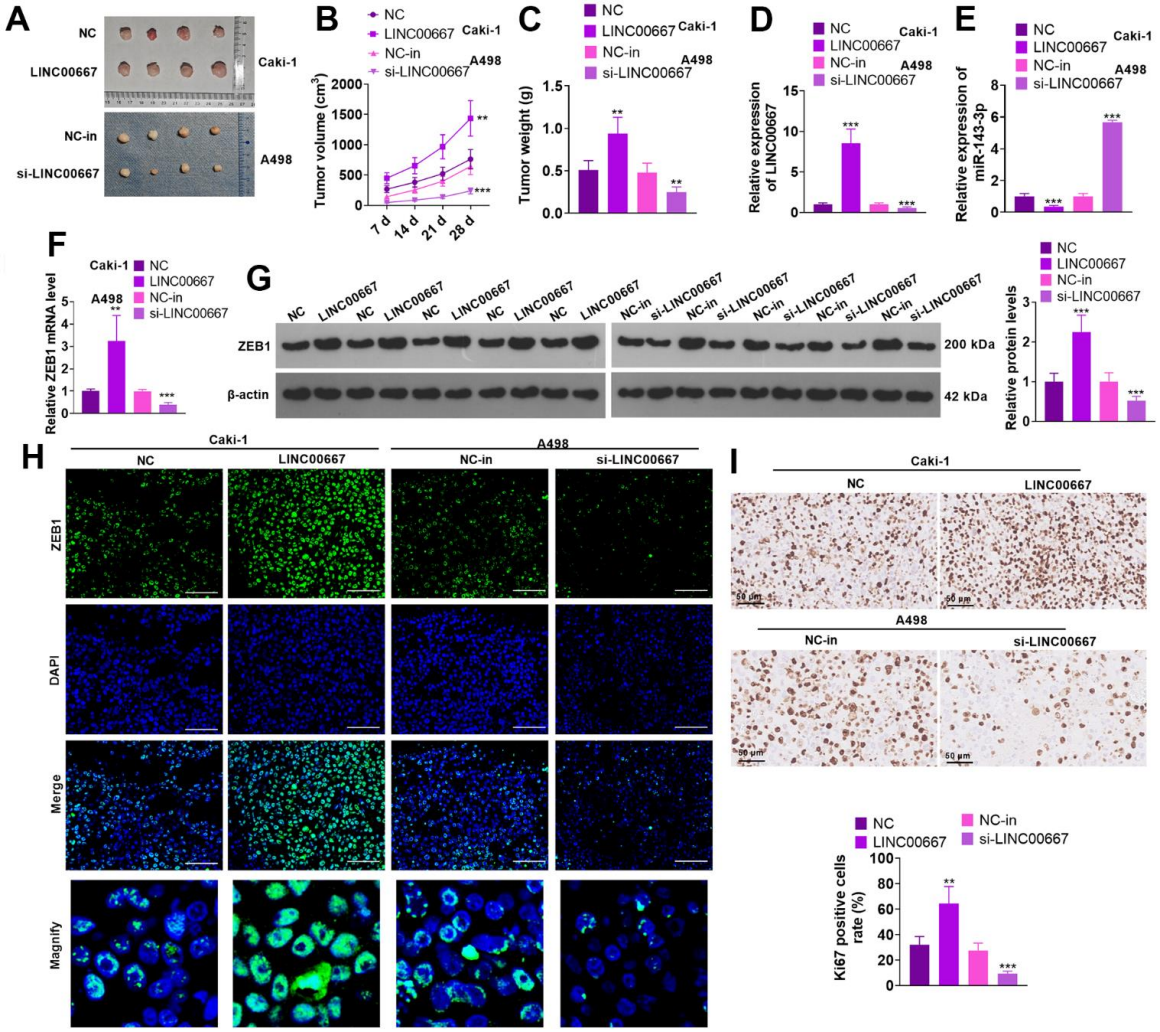
**Regulation of ccRCC tumor growth by LINC00667.** Caki cells or A498 cells with selective regulation of LINC00667 were transfused subcutaneously into the mice to construct *in-vivo* tumor models. (**A**) Plots of tumor tissues were stripped from mice on day 28 after cell injection. (**B**) Changes in the tumor volume. (**C**) The tumor mass. (**D**, **E**) LINC00667 and miR-143-3p levels in tumor tissues were ascertained by qRT-PCR. (**F**) The ZEB1 mRNA level in tumor tissues was gauged via qRT-PCR. (**G**) The ZEB1 protein level in tumor tissues was gauged via WB. (**H**) immunofluorescence was conducted for detecting ZEB1 in the tumor tissues. Scale bar=50 μm. (**I**) Ki67 profile in tumor tissues verified by IHC. The Ki67-positive cell rate was counted. ns*P*>0.05, **P<0.01, ***P<0.001 (vs. NC or NC-in). N=5.

Caki cells (with LINC00667 overexpression) and A498 cells (with LINC00667 knockdown) were applied for constructing *in-vivo* tumor models. As a result, LINC00667 overexpression significantly advanced the growth of tumor volume and mass versus the NC group. In parallel, transfection of LINC00667-in suppressed the increase in the tumor volume and mass versus the NC-in group (Figure 4A–4C, *P*<0.05). qRT-PCR showed that LINC00667 overexpression notably facilitated LINC00667 expression and choked miR-143-3p profile in the tumor tissues (versus NC). By contrast, LINC00667-in significantly reduced LINC00667 expression and augmented miR-143-3p profile (against NC-in) (Figure 4D, 4E, *P*<0.05). qRT-PCR, WB and immunofluorescence results exhibited that overexpressing LINC00667 significantly facilitated ZEB1 expression (vis-à-vis NC). In contrast, LINC00667 knockdown vigorously abated ZEB1 expression versus the NC-in group (Figure 4F–4H, *P*<0.05). Overexpressing LINC00667 greatly enhanced Ki67-positive cell rate, and knocking down LINC00667 reduced Ki67-positive cell rate (Figure 4I, *P*<0.05). Thus, overexpressing LINC00667 accelerated ccRCC cell growth in an *in-vivo* xenograft tumor model.

### The lncRNA-miRNA-mRNA-associated ceRNA regulatory network

**Figure 5:**
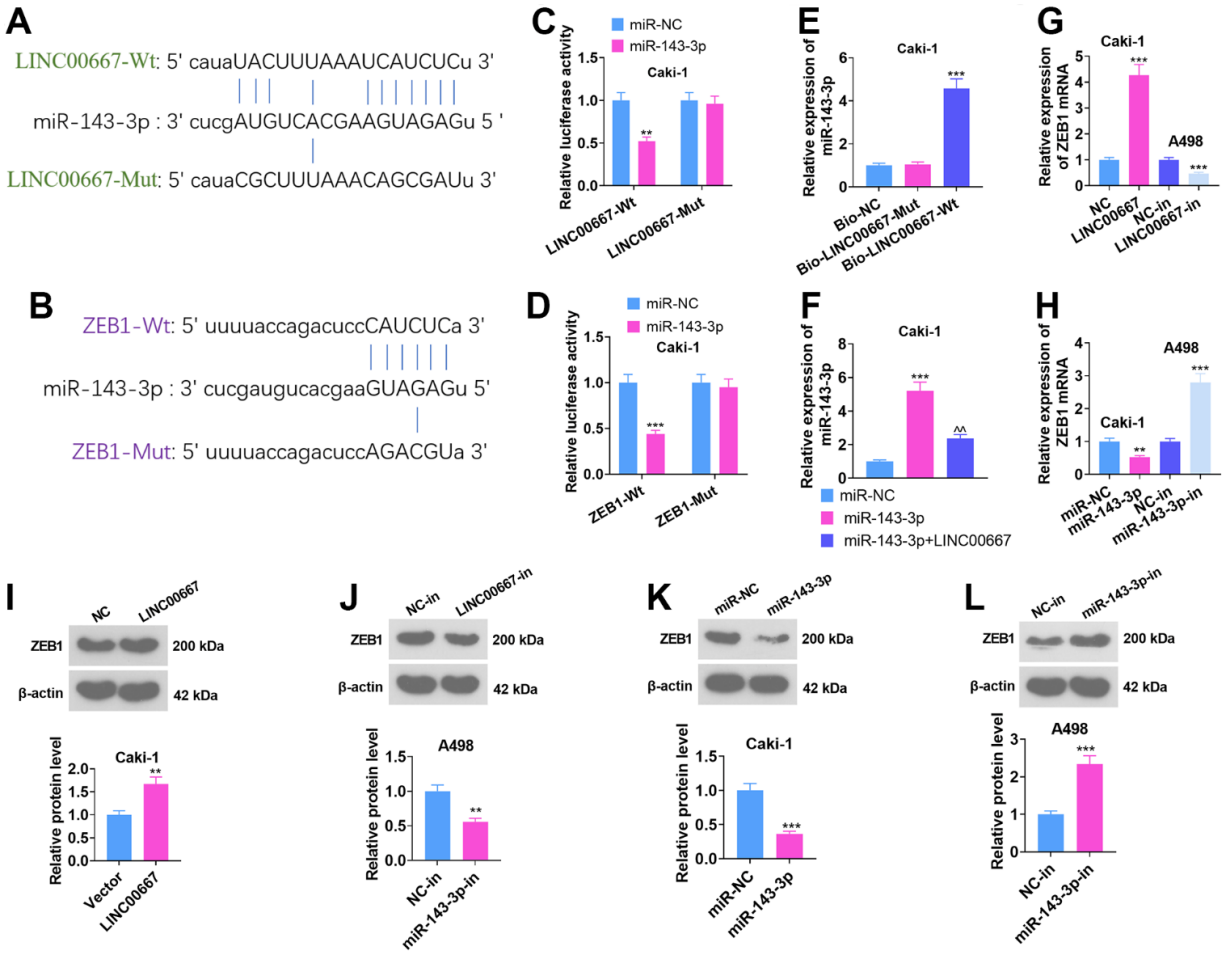
**The LINC00667-miR-143-3p-ZEB1-associated ceRNA regulatory network.** (**A**, **B**) The binding sites between LINC00667 and miR-143-3p and between miR-143-3p and ZEB1 searched through the ENCORI database (starbase.sysu.edu.cn). (**C**, **D**) The influence of miR-143-3p mimics on LINC00667-Wt, LINC00667-Mut, ZEB1-Wt, and ZEB1-Mut luciferase activities were checked using a dual-luciferase reporter assay. (**E**) The impact of biotin-labeled miR-143-3p on LINC00667 monitored by RNA Pull-down assay. (**F**) A498 cells were transfected with miR-NC, miR-143-3p, and miR-143-3p+LINC00667. The miR-143-3p level was gauged via qRT-PCR. LINC00667 overexpression plasmids and miR-143-3p mimics were employed to transfect Caki, while LINC00667-in and miR-143-3p-in were harnessed to transfect A498. (**G**, **H**) The mRNA level of ZEB1 in Caki and A498 was assayed using qRT-PCR. (**I**–**L**) The ZEB1 level in Caki and A498 cells was assessed by WB. **P<0.01, ***P<0.001 (vs. miR-NC/NC/NC-in). ^^*P*<0.01 (vs. miR-143-3p). N=3.

As predicted by ENCORI (starbase.sysu.edu.cn), the base binding sites between LINC00667 and miR-143-3p and between miR-143-3p and ZEB1 were identified (Figure 5A, 5B). Dual-luciferase reporter assay demonstrated overexpressing miR-143-3p substantially dampened LINC00667-Wt and ZEB1-Wt luciferase activities versus the miR-NC group (Figure 5C, 5D, *P*<0.05). RNA Pull-down assay highlighted that LINC00667 was specifically enriched in the miR-143-3p probe assay (vis-à-vis miR-NC) (Figure 5E, *P*<0.05). The outcomes of qRT-PCR illustrated that the miR-143-3p level markedly increased in Caki cells subsequent to miR-143-3p mimics transfection versus the miR-NC group, whereas transfection with the LINC00667 overexpression plasmids distinctly suppressed the miR-143-3p level versus the miR-143-3p group (Figure 5F, *P*<0.05). In addition, LINC00667 overexpression boosted the ZEB1 level and LINC00667-in notably curbed ZEB1 expression versus the NC-in group (Figure 5G, 5I, 5J, *P*<0.05). miR-143-3p mimics repressed ZEB1 expression versus the miR-NC group, while miR-143-3p-in notably upregulated ZEB1 profile versus the NC-in group (Figure 5H, 5K, 5L, *P*<0.05). The above results suggested a targeted correlation between LINC00667 and miR-143-3p, as well as between miR-143-3p and ZEB1.

### The regulation of miR-143-3p-ZEB1 axis on the biological functions of ccRCC cells

**Figure 6:**
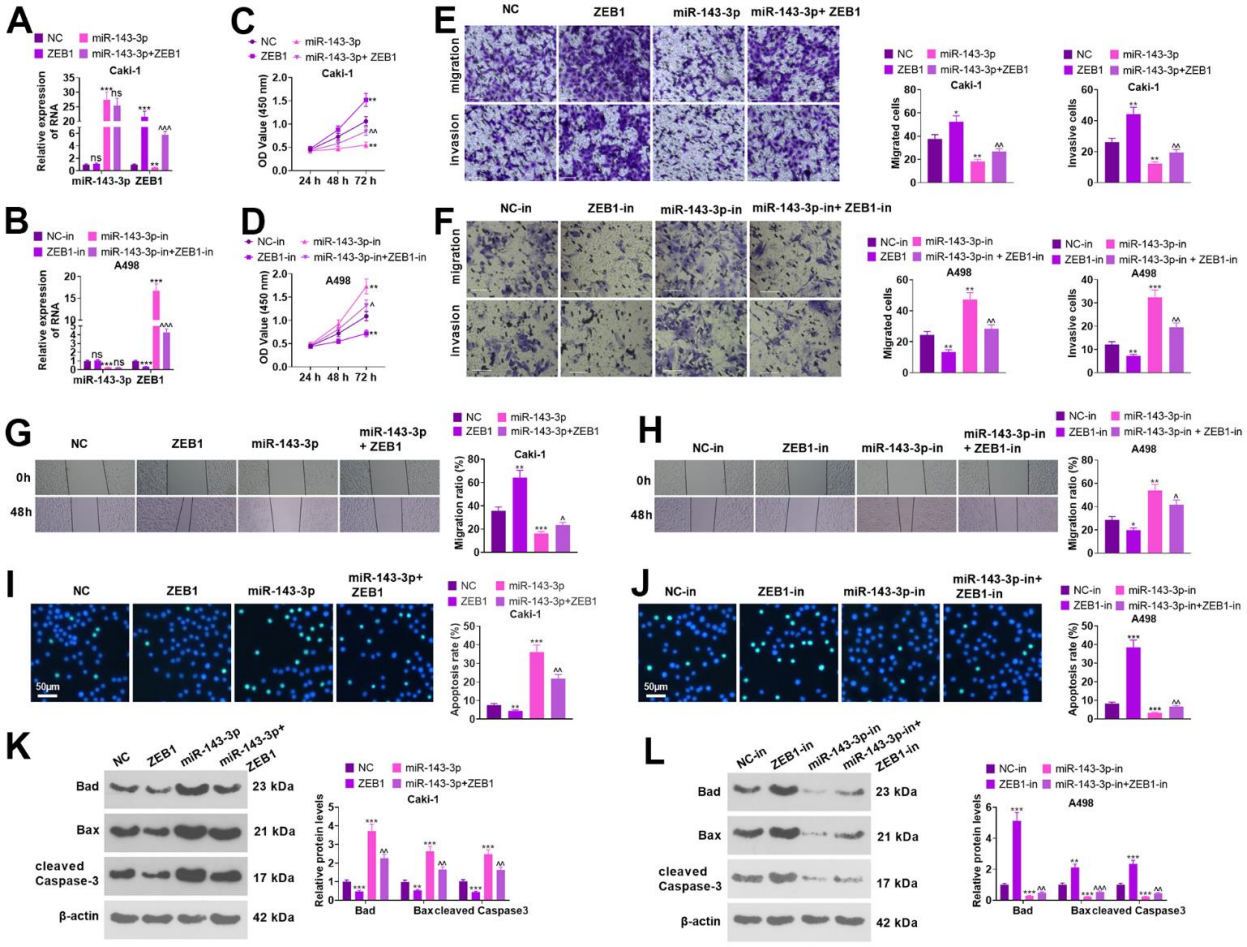
**Regulation of biological functions of ccRCC cells by miR-143-3p-ZEB1 axis.** Caki cells were transfected together with ZEB1 overexpression plasmids and/or miR-143-3p mimics, and A498 cells were transfected along with ZEB1-in and/or miR-143-3p-in. (**A**, **B**) miR-143-3p and ZEB1 levels in Caki and A498 cells verified by employing qRT-PCR. (**C**, **D**) Cell proliferation was examined using CCK-8. (**E**, **F**) Migration and invasion in the cells checked by Transwell. Scale bar=100 μm. (**G**, **H**) The migration of the cells was evaluated by the wound healing test. (**I**, **J**) Cell apoptosis monitored using TUNEL. Scale bar=50 μm. (**K**, **L**) The levels of apoptosis-associated proteins were compared using WB. ns*P*>0.05, **P<0.01, ***P<0.001 (vs. NC or NC-in). ns*P*>0.05, ^^*P*<0.01, ^^^*P*<0.001 (vs. miR-143-3p /miR-143-3p-in). N=3.

Caki cells were transfected together with ZEB1 overexpression plasmids and/or miR-143-3p mimics, and A498 cells were transfected with ZEB1-in and/or miR-143-3p-in (Figure 6A, 6B, *P*<0.05). Functional assays showed that overexpressing miR-143-3p choked the proliferation, migration, and invasion activities of Caki cells versus the NC group. In parallel, overexpression of ZEB1 significantly heightened proliferation, migration and invasion in Caki cells (in contrast with NC or miR-143-3p) (Figure 6C, 6E, 6G, *P*<0.05). miR-143-3p inhibition resulted in enhanced levels of migration, invasion, and proliferation in A498 cells versus the NC-in group. ZEB1-in notably curbed the activities of proliferation, migration and invasion in A498 cells versus the NC-in or miR-143-3p-in group (Figure 6D, 6F, 6H, *P*<0.05). As shown by the WB data, overexpressing ZEB1 or repressing miR-143-3p remarkably reduced cell apoptosis and enhanced EMT. Oppositely, ZEB1 knockdown or miR-143-3p upregulation promoted apoptosis and inhibited EMT (Figure 6I–6L, *P*<0.05). Thus, the miR-143-3p-ZEB1 axis greatly affected the apoptosis, migration, invasion, and proliferation of ccRCC cells.

### The regulation of ccRCC cells’ biological functions by LINC00667-miR-143-3p-ZEB1 axis

**Figure 7:**
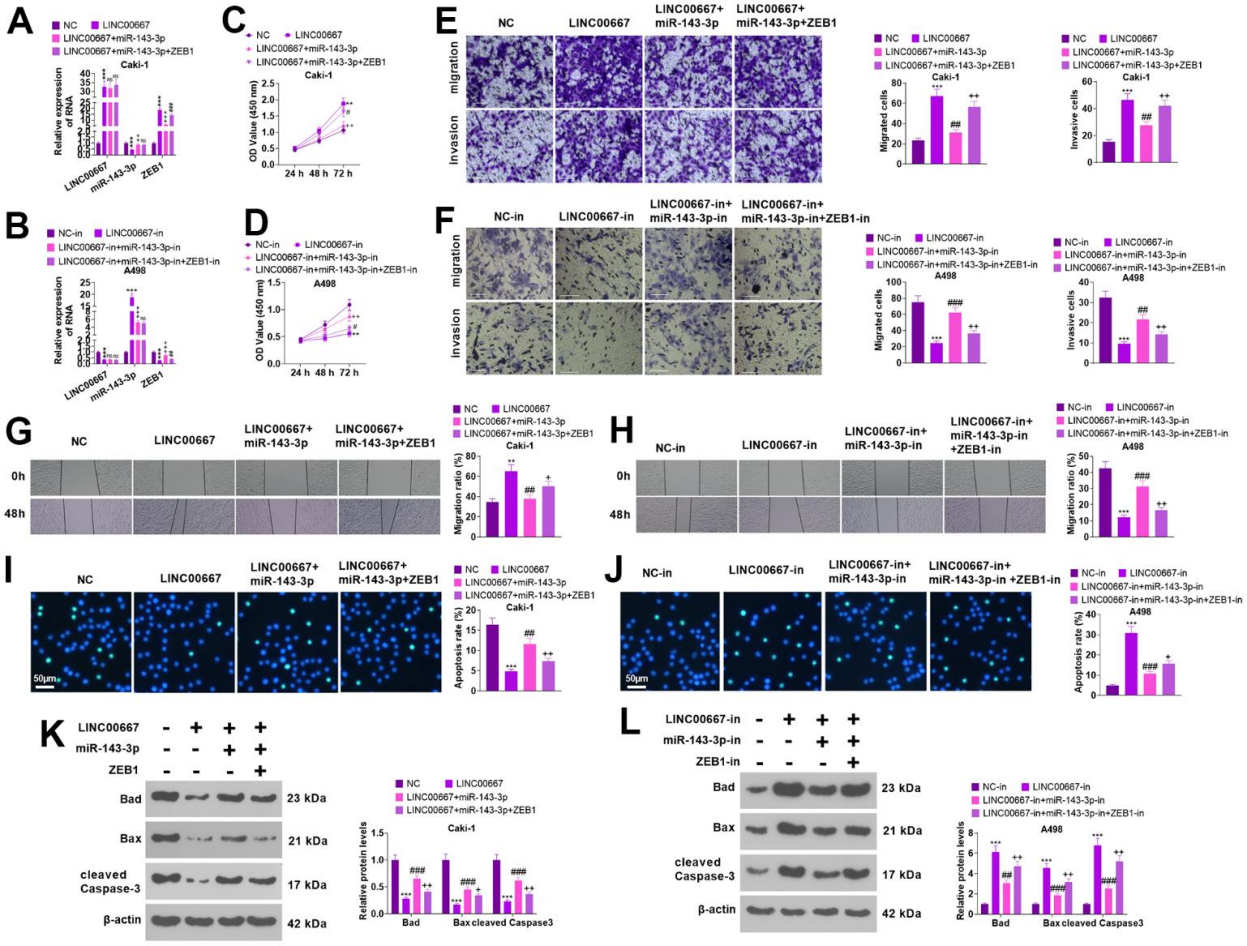
**The regulation of the biological function of ccRCC cells by LINC00667-miR-143-3p-ZEB1 axis.** Caki cells were transfected along with LINC00667 overexpression plasmids, miR-143-3p mimics and/or ZEB1 overexpression plasmids, while A498 cells were transfected together with LINC00667-in, miR-143-3p-in, and/or ZEB1-in. (**A**, **B**) LINC00667, miR-143-3p, and ZEB1 levels in Caki and A498 cells were tested via qRT-PCR. (**C**, **D**) Cell proliferation verified by CCK-8. (**E**, **F**) Migration and invasion in the cells were checked through Transwell. (**G**, **H**) Cell migration monitored by the wound healing test. Scale bar=100 μm. (**I**, **J**) Apoptosis gauged through TUNEL assay. Scale bar=50 μm. (**K**, **L**) The levels of apoptosis-concerned proteins were ascertained by WB. **P<0.01, ***P<0.001 (vs. NC/NC-in). ns*P*>0.05, ^^*P*<0.01, ^^^P<0.001 (vs. LINC00667 or LINC00667-in). ns*P*>0.05, #*P*<0.05, ##*P*<0.01, ###*P*<0.001 (vs. LINC00667+miR-143-3p or LINC00667-in+miR-143-3p-in). N=3.

Rescue assays were conducted for to verify the functions of the LINC00667-miR-143-3p-ZEB1 axis in tumor cells (Figure 7A, 7B, *P*<0.05). As a result, miR- 143-3p repressed LINC00667 induced cell proliferation, migration, and invasion elevation, and enhanced cell apoptosis. Subsequent to ZEB1 overexpression, the migration, invasion, and proliferation levels of Caki went up, whereas apoptosis decreased (vis-à-vis LINC00667+miR-143-3p) (Figure 7C, 7E, 7G, 7I, 7K *P*<0.05). Knocking down miR-143-3p visibly reversed the LINC00667 knockdown-mediated tumor-suppressive effect versus the LINC00667-in group. Compared with LINC00667-in+miR-143-3p-in, knocking down ZEB1 culminated in significantly lessened proliferation, migration, and invasion and enhanced apoptosis in A498 (Figure 7D, 7F, 7H, 7J, 7L, *P*<0.05). Thus, overexpressing LINC00667 boosted ZEB1 by down-regulating miR-143-3p, which in turn fostered migration, invasion, and proliferation in ccRCC cells and restrained their apoptosis.

### Regulatory mechanisms of lncRNA-miRNA-mRNA in EMT

**Figure 8:**
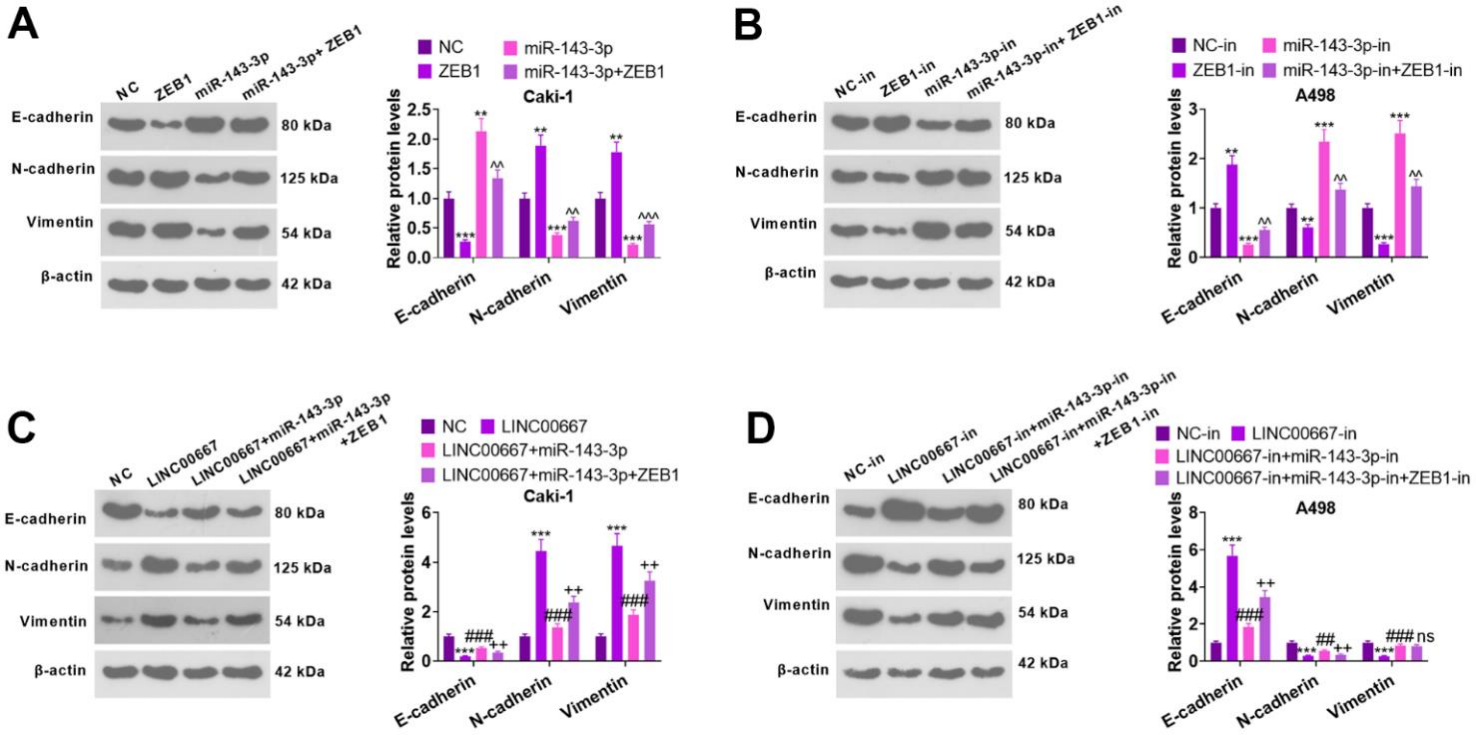
**The regulatory mechanism of LINC00667-miR-143-3p-ZEB1 axis on EMT.** Caki cells were transfected using ZEB1 overexpression plasmids and/or miR-143-3p mimics, whereas A498 cells were transfected employing ZEB1-in and/or miR-143-3p-in. (**A**, **B**) The profiles of EMT markers tested by WB. [**P<0.01, ***P<0.001 (vs. NC or NC-in). ^^*P*<0.01, ^^^*P*<0.001 (vs. miR-143-3p/miR-143-3p-in)]. Caki cells were transfected with the addition of LINC00667 overexpression plasmids, miR-143-3p mimics, and/or ZEB1 overexpression plasmids, while A498 cells were transfected together with LINC00667-in, miR-143-3p-in and/or ZEB1-in. (**C**, **D**) The profiles of EMT markers were ascertained via WB. [***P<0.001 (vs. NC/NC-in). ^^*P*<0.01, ^^^*P*<0.001 (vs. LINC00667 or LINC00667-in). ns *P*>0.05, ##*P*<0.01 (vs. LINC00667+miR-143-3p or LINC00667-in+miR-143-3p-in). N=3.

WB checked E-cadherin, N-cadherin, and Vimentin levels (Figure 8A–8D). As a result, overexpressing miR-143-3p elevated the E-cadherin level and repressed N-cadherin and Vimentin contents (versus NC). Vis-à-vis NC or miR-143-3p, overexpressing ZEB1 distinctly restricted E-cadherin’s level and stimulated N-cadherin and Vimentin levels (Figure 8A, *P*<0.05). miR-143-3p-in transfection brought about a distinct decrease in the level of E-cadherin and a marked increase in N-cadherin and Vimentin profiles (versus the NC-in group). In contrast, transfection with ZEB1-in remarkably facilitated the profile of E-cadherin and diminished N-cadherin and Vimentin levels (against NC-in or miR-143-3p-in) (Figure 8B, *P*< 0.05). As compared with NC, overexpressing LINC00667 choked the E-cadherin expression level and drove up N-cadherin and Vimentin profiles. On the contrary, overexpressing miR-143-3p heightened the level of E-cadherin and dampened N-cadherin and Vimentin expressions (versus LINC00667). Meanwhile, substantially impeded E-cadherin expression and bolstered N-cadherin and Vimentin levels were observed following ZEB1 overexpression (versus LINC00667+miR-143-3p) (Figure 8C, *P*<0.05). LINC00667-in greatly expanded E-cadherin expression and suppressed N-cadherin and Vimentin levels (vis-a- vis NC-in). Compared to the LINC00667-in group, transfection with miR-143-3p-in brought about a remarkable reduction in the profile of E-cadherin and a significant decrease in N-cadherin and Vimentin levels. Knockdown of ZEB1 dramatically facilitated E-cadherin’s expression and lessened the N-cadherin profile (against LINC00667-in+miR-143-3p-in) (Figure 8D, *P*<0.05). These results corroborated that overexpressing LINC00667 facilitated the EMT of ccRCC via the miR-143-3p- ZEB1 axis.

## DISCUSSION

In this study, we explored the expression features and functions of LINC00667 in ccRCC. Our experiment both *in vitro* and *in vivo* suggested that LINC00667 aggravated the malignant behaviors of ccRCC cells and promoted DDP resistance. These studies implied that LINC00667 displayed carcinogenic functions in ccRCC development.

LINC00667 takes part in regulating the evolvement of multiple tumors. For instance, LINC00667 stimulates migration, invasion, and proliferation in cells pertaining to esophageal cancer by mediating the sponge axis miR-200b-3p/SLC2A3 [19]. LINC00667 up-regulates FOXQ1 by combining with miR-4319 in a competitive manner, hence facilitating the malignant phenotype of nasopharyngeal carcinoma cells [20]. It was found that LINC00667 silencing restrained the proliferation, migration and angiogenesis of non-small cell lung cancer cells *ex vivo*. Mechanistic studies have established that the carcinogenic effect of LINC00667 is achieved by stabilizing VEGFA messenger RNA through the recruitment of eukaryotic translation initiation factor 4A3 (EIF4A3) [21]. Here, we found LINC00667 presented a high-level profile in ccRCC cells and tissues. Additionally, overexpressing LINC00667 contributed to ccRCC cell migration, invasion, and proliferation, choked apoptosis, and weakened ccRCC cell sensitivity to DDP. *In-vivo* experiments confirmed that overexpression of LINC00667 expanded tumor growth, which was generally consistent with previous findings.

It has been strongly evidenced that lncRNAs competitively occupy the shared binding sequences of miRNAs by acting as ceRNAs, thereby isolating miRNAs and affecting the downstream target gene level [22–24]. The ceRNA network formed by lncRNA/miRNA/mRNA interactions has been identified in various cancers. For example, lncRNA-CDC6 shows a high-level profile in breast cancer tumor tissues and positively correlates with the clinical stage of breast cancer patients. lncRNA-CDC6 elevates CDC6 expression by directly sponging miR-215 to function as a miRNA sponge, which boosts proliferation and metastasis in breast cancer-related cells [25]. Another research unravels that LINC00355 expression is heightened in bladder cancer tissues and that highly-expressed LINC00355 contributes to poor prognosis in bladder cancer patients. In-depth probing discloses that LINC00355 up-regulates HMGA2 by sponging miR-424-5p, thereby heightening migration, invasion, and EMT of bladder cancer cells and facilitating lung metastasis of xenograft tumors [26]. Here, bioinformatics analysis revealed a binding site between LINC00667 and miR-143-3p. miR-143-3p has been substantiated to suppress the progression of ovarian cancer [27], colorectal cancer [28] and non-small cell lung cancer [29] in several previous studies. Meanwhile, dual-luciferase reporter and RNA pull-down assays both identified the targeted correlation between LINC00667 and miR-143-3p. Besides, overexpressing miR-143-3p abated the carcinogenic effects mediated by LINC00667 in ccRCC, suggesting that LINC00667 accelerated ccRCC progression by inhibiting miR-143-3p in a targeted manner.

The high-level profile of Zinc-finger E-box binding protein 1 (ZEB1), a transcription factor, is strongly linked to cancer malignancy. New evidence reveals that ZEB1 drives EMT and thus advances tumor invasion and metastasis by initiating stem cell features, immune evasion, and epigenetic reprogramming [30, 31]. ZEB1 is up-regulated in osteosarcoma, cervical, and hepatocellular carcinoma cells, contributing to the malignant phenotype of tumor cells. It is reportedly a downstream target of miR-143-3p [32–34]. Similarly, given the base-pairing association between miR-143-3p and ZEB1, we revealed through dual-luciferase reporter assay and qRT-PCR that miR-143-3p targeted ZEB1 and choked ZEB1 expression in ccRCC cells. Notably, studies have identified a regulatory role for ZEB1 in ccRCC. For example, lncRNA SNHG5 augments the ZEB1 level by sponging miR-205-5p, which in turn fosters ccRCC cells’ proliferation, migration and invasion, tumorigenesis and metastasis [35]. Furthermore, the profile of miR-508 was dramatically lowered in the tissues and cells of ccRCC.

Overexpressing miR-508 markedly diminished the invasion and proliferation levels of ccRCC cells. ZEB1 was demonstrated as a direct target gene for miR-508 in ccRCC cells. The relative profile of ZEB1 mRNA was upped in ccRCC tissues. Both ZEB1 knockdown and miR-508 overexpression mediated tumor-suppressive effects on ccRCC cells [36]. Here, we determined that overexpression of ZEB1 fostered proliferation, migration, invasion, and EMT in ccRCC cells and retarded their apoptosis. Besides, overexpressing ZEB1 abated the inhibitory influence of miR-143-3p on ccRCC cells.

However, several shortcomings should be further investigated in the future. First, the present study mainly explored gene regulation in two ccRCC cell lines (Caki and A498), and functional assays should be performed in more ccRCC cells for verifying the role of the LINC00667-miR-143-3p-ZEB1 axis in ccRCC progression. Second, more clinical samples from ccRCC patients should be collected for evaluating the diagnostic role of the above axis in ccRCC. Third, it’s valuable to test the role of the LINC00667-miR-143-3p-ZEB1 axis in ccRCC cells treated with more chemotherapy drugs.

## CONCLUSIONS

Overall, our research confirmed that LINC00667 expression was fostered in ccRCC cells and tissues and that overexpressing LINC00667 boosted ccRCC cell growth both *ex vivo* and *in vivo*. Importantly, we confirmed a new ceRNA regulatory axis in which LINC00667 sponged miR-143-3p so as to up-regulate ZEB1. Hence, LINC00667 is a potential target for treating ccRCC patients.

## MATERIALS AND METHODS

### Collection and processing of clinical specimens

Fifty-two tumor specimens and corresponding non-tumor tissues were harvested from the patients who went through ccRCC surgery in the Affiliated Hospital of Hebei Engineering University (from July 2018 to March 2019) as the study subjects. The histological and pathological diagnosis of the specimens was corroborated by two senior pathologists by utilizing the 2016 World Health Organization classification and staging system for renal tumors and the Fuhrman classification. All specimens were subject to the patient’s informed consent. The Ethical Committee of the Affiliated Hospital of Hebei Engineering University authorized the experiment (Approval number: 2018-022-14). The specific information of the patients is shown in Table 1. After the operation, the tissue specimens were refrozen in liquid nitrogen in preparation for the following trials.

### Culture of cells

The American Typical Culture Collection (ATCC, Manassas, VA, USA) provided us with the cells: Hkb20, human renal epithelial cells, as well as Caki-1, SN12-PM6, RCC4, A498, A704, and SW839 (ccRCC cell lines). A DMEM medium was taken to grow Hkb20 cells. A McCoy’s 5A medium was exploited to cultivate Caki-1 cells. In the 89% McCoy’s 5A medium cultivated SN12-PM6 cells. RCC4 cells were cultivated with 90% DMEM (high sugar). A498 and A704 cells were cultured with MEM medium. An RPMI 1640 medium was harnessed to grow SW839 cells. All these culture media comprised high-quality fetal bovine serum (10%). The incubation was implemented under the conditions of 37° C and 5% CO_2_. We changed the media every 1-2 days. When 80-90% of the bottle bottom was covered, 0.25% trypsin was harnessed to trypsinize the cells. The above-mentioned media were bought from Thermo Fisher Scientific (Shanghai, China).

### Cell transfection

What we ordered from GenePharma Co., Ltd. (Shanghai, China) included pcDNA-LINC00667 (LINC00667), small interference-LINC00667 (si-LINC00667), miR-143-3p mimics, miR-143-3p inhibitors (miR-143-3p-in), pcDNA-ZEB1 (ZEB1) and Si-ZEB1, pcDNA empty vectors (NC) and small interference-normal control (NC-in) and miRNA control (miR-NC). Prior to the transfection of cells, Caki-1 and A498 cells, inoculated onto 24-well plates (3×10^5^ cells/well), were kept at 37° C under the conditions of 5% CO_2_ for 24 hours. As indicated by the supplier, Lipofectamine® 3000 (Thermo Fisher Scientific, Shanghai, China) was exploited to transfect Caki-1 and A498. qRT-PCR validated the transfection. The cells were subjected to a 24-hour incubation with 5% CO_2_ at 37° C in preparation for further analysis.

### qRT-PCR

TRIzol reagent (Invitrogen, Carlsbad, CA, USA) isolated the total RNA from the cells and tissues. Thermo NanoDrop 2000 checked the RNA concentration and purity. With the help of the ReverTra Ace qPCR RT Master Mix with gDNA Remover kit (Toyobo, Japan) or the commercial miRNA reverse-transcription PCR kit (GenePharma, Shanghai, China), the RNA was reversely transcribed into cDNA. With the help of SYBR Premix Ex Taq II (Takara, Japan), we performed qRT-PCR on the system of Mx3005P real-time PCR (Stratagene, San Diego, CA, USA). GAPDH was selected as the endogenous control for lncRNA and mRNA, while U6 served as that of miRNA. The reaction conditions were 30 seconds of pre-denaturation at 95° C, 5 seconds of denaturation at 95° C, and 30 seconds of annealing at 60° C, with 45 cycles in total. The target and internal reference genes were amplified for each sample. Each group encompassed 3 replicate wells. The relative profiles of the target genes were analyzed using the 2^-ΔΔCT^ method. The primer sequences for each target gene are exhibited in Table 2.

**Table 2:** The sequences of primers.

**Primer**	**Forward (5’ to 3’)**	**Reverse (5’ to 3’)**
LINC00667	CATGGGCTGGTATGAGTTGC	TTGGCTGGGATCTCACACAT
miR-143-3p	GGGGTGAGATGAAGCACTG	CAGTGCGTGTCGTGGAGT
ZEB1	CTCTTCAGGTGCCTCAGGAA	CAGGGAGGAGCAGTGAAAGA
GAPDH	TGGTTGAGCACAGGGTACTT	CCAAGGAGTAAGACCCCTGG
U6	CCTGCGCAAGGATGAC	GTGCAGGGTCCGAGGT

### Dual-luciferase reporter gene assay

Dual-luciferase reporter gene assay was done with the dual-luciferase reporter assay system (Promega, Madison, WI, USA). The wild-type plasmids (LINC00667-Wt and ZEB1-Wt) and mutant plasmids (LINC00667-Mut and ZEB1-Mut) were designed by GeneScript Co., Ltd. (Nanjing, China). LINC00667-Wt, LINC00667-Mut, ZEB1-Wt or ZEB1-Mut were transfected together with miR-143-3p mimics or miR-NC into Caki-1 cells. The activities of firefly luciferase and renilla luciferase were gauged 48 hours subsequent to transfection. These experiments were implemented in triplicate and conducted 3 times.

### RNA pull-down assay

Genepharma (Shanghai, China) supplied us with purified biotin-labeled LINC00667-Wt and LINC00667-Mut, which were transfected into Caki-1 cells for a period of 48 hours. In line with the supplier’s guidance, Dynabeads™ M-280 Streptomycin Affinity (Thermo Fisher Scientific, Waltham, MA, USA) was harnessed to maintain the cell lysates. TRIzol eluted and purified the interacting RNA compounds. qRT-PCR verified the miR-143-3p level.

### CCK-8 assay

A498 and Caki-1 were inoculated onto 96-well plates (2×10^3^ cells/well). Subsequent to 24-hour adherent culture, each well was filled with 10 μL CCK-8 (MedChem Express, Monmouth Junction, NJ, USA) solution. The plates were maintained for 4 hours. The absorbance values (450 nm) were gauged by employing a microplate reader as a means of determining alterations in A498 and Caki-1 cell proliferation at the 24^th^, 48^th,^ and 72^nd^ hour following transfection and co-transfection, respectively.

### Transwell assay

Migration and invasion of tumor cells were checked through Transwell. In the upper Transwell chambers, A498 and Caki-1 cells (2×10^4^ cells/well) were seeded. The lower chambers contained 600 μL of a culture medium comprising FBS (20%). The cells went through incubation at 37° C. Twelve hours later, we removed the cells present in the upper compartment. They were frozen in paraformaldehyde (4%), dyed with 0.1% crystalline violet, air-dried, photographed, and counted. The cell invasion experiment was done in the same way as the experiment of migration, barring that the upper Transwell chamber was pre-coated using Matrigel (8 μM pore-size; Corning, Beijing, China) before the cells were added.

### Wound healing test

A498 and Caki-1 cells at logarithmic growth were inoculated onto 6-well culture plates (1×10^6^/mL). Upon 80% to 90% fusion of the cells, a thin scratch was made perpendicular to the cells with a sterile pipette. PBS was adopted to remove floating cells three times. A DMEM medium supplemented with 2.5% fetal bovine serum (Thermo Fisher Scientific, Shanghai, China) was added and kept for 24 hours (37° C). The cells were immobilized in 4% paraformaldehyde, and the migration capacity was indicated microscopically by measuring the width of the scratch.

### TUNEL assay

Following stable transfection, we harvested the cells of A498 and Caki-1. Prior to the removal of the medium, the cells were cultivated for another 24 hours. PBS flushed the cells, which were then fastened with 4% paraformaldehyde (15 min) and permeabilized employing 0.25% Triton-X 100 (20 min). The TUNEL assay solution (50 μL) was given for incubating the samples in the dark (60 min; 37° C). PBS flushed the cells 3 times. An anti-fluorescence quenching sealing solution was utilized for sealing. A fluorescent microscope with excitation light at 450-500 nm and emission light (green fluorescence) at 515-565 nm was employed for observation. Three fields of view were picked randomly for each sample to check apoptosis. Apoptotic rate = apoptotic cell number/total cell number × 100%.

### Western blot

With the use of RIPA lysis buffer (Beyotime Biotechnology, Shanghai, China), the total protein was separated from the cells or tissues. The Bradford method enabled us to confirm the protein concentration. Protein samples of the comparable amount were taken for 12% sodium dodecyl sulfate-polyacrylamide gel electrophoresis (SDS-PAGE) (Thermo Fisher Scientific, Shanghai, China) and then transferred onto polyvinylidene difluoride (PVDF) membranes (Millipore, Bedford, MA, USA). Next, 5% skimmed milk powder was added to seal the membranes at RT for 2 hours. What followed was the overnight incubation of the membranes at 4° C with the primary antibodies mentioned here: Anti-Bad (ab32445, 1:1000), Anti-Bax (ab32503, 1:1000), Anti-cleaved Caspase-3 (ab32042, 1:1000), Anti-ZEB1 (ab203829, 1:1000), Anti-E-cadherin (ab40772, 1:1000), Anti-N-cadherin (ab76011, 1:1000), Anti-Vimentin (ab92547, 1:1000), Anti-p-c-Jun (ab32385, 1:1000), Anti-c-Jun (ab40766, 1:1000), Anti-p-JNK1 (ab501858, 1:1000), Anti-JNK1 (ab38050, 1:1000), Anti-JAK1 (ab133666, 1:1000), Anti-p-STAT3 (ab68153, 1:1000), Anti-STAT3 (ab32143, 1:1000), and Anti-β-actin (ab8227, 1:1000). Following washes with TBST, the secondary antibody labeled with HRP (ab6721, 1:1000) was added for 2-hour incubation at RT. TBST cleaned the membranes three times. At last, visualization was performed employing ECL (Millipore, Bedford, MA, USA). β-actin was chosen as the endogenous control protein. Primary antibodies p-JNK1 and JNK1 were supplied by Abcam (Cambridge, UK), and the remaining antibodies were provided by Biorbyt (Cambridge, UK).

### Tumor formation experiment

Forty nude mice on a BALB/c background were acquired from the Animal Experiment Center of Hebei Engineering University. They were divided into eight groups (five mice each). Caki-1 (transfected with NC or LINC00667) and A498 (transfected along with NC-in or si-LINC00667) were made into cell suspensions (concentration: 2×10^7^ cells/mL), which were then transfused subcutaneously into the nude mice (0.1 mL/ mouse). Vernier calipers gauged the tumors’ long and short diameters. As per the formula (V= 0.5×long diameter× short diameter^2^), the tumor volume (V) was worked out. Four weeks after administration, all the animals were euthanized, with their tumor tissues surgically removed and weighed. All experimental procedures abided by the instructions for the use and care of laboratory animals.

### Tissue immunofluorescence

Tumor tissues were gathered, frozen and sectioned (15 μM thick). Subsequently, the sections were sealed using 10% goat serum at 37° C for 2 hours and then maintained with the primary antibody ZEB1 (ab203829, 1:250, Abcam) overnight at 4° C. Next, the slices were flushed 3 times in PBS. The secondary antibody goat anti-rabbit IgG (ab150077, 1:500, Abcam) was given for 2-hour incubation at 37° C. PBS flushed the slices 3 times, which were then sealed by a sealer containing DAPI (Beyotime, Shanghai, China). The photographs were taken with a confocal microscope (Zeiss, Germany). Three sections of the tumor tissues of each rat were stained at random. Photographs were taken in five fields of view randomly selected from each slice.

### Immunohistochemistry (IHC)

First of all, the tissues were embedded in paraffin and sliced up. After dewaxing and hydration, the slices went through high-pressure repair for 15 minutes with the use of 0.01 mmol/L sodium citrate buffer solution and naturally cooled. 3 mL/L of H_2_O_2_ was added dropwise to the wet box and kept for 10 minutes to eliminate the activity of endogenous peroxidase. After the gradual addition of normal goat serum blocking solution, the primary antibody Anti-Ki67 (ab92742, 1:100, Abcam) was added and maintained with the sections overnight at 4° C. Subsequent to PBS rinsing, Goat Anti-Rabbit IgG Antibody (ab97051, 1:200) was utilized for 30-minute incubation at RT. PBS rinsed the slices. DAB was adopted for 3 minutes of color development. Hematoxylin redyed the nuclei. Prior to microscopic examination, the cells were dehydrated, made transparent, and blocked.

### Analysis of statistics

GraphPad Prism 8 (GraphPad Software, Boston, MA, USA), a software designed for statistical analysis, analyzed the experimental outcomes, which were presented as mean ± standard deviation. Data with uniform variance and normal distribution were contrasted through a one-way analysis of variance (ANOVA) or Student’s t-test, or conversely, using a rank-sum test. If the P value was below 0.05, statistical significance was identified.

### Data availability statement

The data sets used and analyzed during the current study are available from the corresponding author upon reasonable request.
